# A unique population of effector memory lymphocytes identified by CD146 having a distinct immunophenotypic and genomic profile

**DOI:** 10.1186/1471-2172-8-29

**Published:** 2007-11-13

**Authors:** Mohamed F Elshal, Sameena S Khan, Nalini Raghavachari, Yoshiyuki Takahashi, Jennifer Barb, James J Bailey, Peter J Munson, Michael A Solomon, Robert L Danner, J Philip McCoy

**Affiliations:** 1 Flow Cytometry Core Facility, National Heart, Lung, and Blood Institute, National Institutes of Health. Bethesda, MD, USA; 2 Functional Genomics and Proteomics Facility, Critical Care Medicine Department, Clinical Center, National Institutes of Health, Bethesda, MD, USA; 3 Hematology Branch, National Heart, Lung, and Blood Institute, National Institutes of Health. Bethesda, MD, USA; 4 Mathematical & Statistical Computing Laboratory, Center for Information Technology, USA; 5 Cardiovascular Branch, National Heart, Lung, and Blood Institute, National Institutes of Health, Bethesda, MD, USA; 6 Molecular Biology Department, Genetic Engineering and Biotechnology Institute, Minoufiya University, Minoufiya, Egypt

## Abstract

**Background:**

CD146 is a well described homotypic adhesion molecule found on endothelial cells and a limited number of other cell types. In cells from the peripheral circulation, CD146 has also been reported to be on activated lymphocytes *in vitro *and *in vivo*. The function associated with CD146 expression on lymphoid cells is unknown and very little information is available concerning the nature of CD146+ lymphocytes. In the current study, lymphocytes from healthy donors were characterized based upon the presence or absence of CD146 expression.

**Results:**

CD146 was expressed on a low percentage of circulating T lymphocytes, B lymphocytes, and NK cells in healthy individuals. CD146 expression can be induced and upregulated *in vitro *on both B cells and T cells, but does not correlate with the expression of other markers of T cell activation. CD146 positive T cells do not represent clonal expansions as determined with the use of anti Vβ reagents. Data suggest that CD146 positive cells have enhanced adherence to endothelial monolayers in vitro. Gene profiling and immunophenotyping studies between CD146+ and CD146- T cells revealed several striking genotypic distinctions such as the upregulation of IL-8 and phenotypic differences including the paucity of CCR7 and CD45RA among CD146 positive T cells, consistent with effector memory function. A number of genes involved in cell adhesion, signal transduction, and cell communication are dramatically upregulated in CD146+ T cells compared to CD146- T cells.

**Conclusion:**

CD146 appears to identify small, unique populations of T as well as B lymphocytes in the circulation. The T cells have immunophenotypic characteristics of effector memory lymphocytes. The characteristics of these CD146+ lymphocytes in the circulation, together with the known functions in cell adhesion of CD146 on endothelial cells, suggests that these lymphocytes may represent a small subpopulation of cells primed to adhere to the endothelium and possibly extravasate to sites of inflammation.

## Background

CD146, a well described marker of endothelial cells, is a cell surface adhesion molecule involved in homotypic and heterotypic cell interactions ([1,2], reviewed by Shih [3]). On endothelial cells, it is located primarily, but not exclusively, at the endothelial junction [4]. Brief reports have also indicated the presence of CD146 on lymphocytes [5-7] and only a single report describes the presence of CD146 on peripheral blood lymphocytes [7]. While widely studied on endothelial cells, very little is known about the presence of CD146 on peripheral blood lymphocytes. Pickl and colleagues reported that CD146 expression could be induced on T cells by mitogen activation in vitro, but failed to detect circulating CD146+ T lymphocytes in healthy individuals[5]. Seftalioglu and Karakoc [6] demonstrated membrane staining of immature thymocytes, macrophages, and epithelial cells using light and electron microscopy in freshly isolated, normal thymic tissue from children, and concluded that CD146 might be a pan-antigen essential for the maintenance of thymic architecture and function. Their observation of the selective expression of CD146 on contact areas of epithelial cells and immature thymocytes led them to conclude that CD146 may play a role in signaling interactions between these two cell types.

In a recent letter, our group reported, for the first time, that a small percentage of lymphocytes freshly isolated from peripheral blood of healthy individuals express cell surface CD146 [7]. These data reveal a small, but consistently demonstrable, subpopulation of lymphocytes which are either genetically pre-programmed to express this antigen, or are activated *in vivo *in a manner that results in this expression. Why such a population would exist in healthy individuals is currently unknown and the subject of current investigation.

CD146 is considered a member of the immunoglobulin gene superfamily and has been reported to show structural sequence similarities with a subgroup of adhesion molecules including the activated lymphocyte cell adhesion molecule CD166 (ALCAM) [3]. In endothelial cells, a number of functional roles have been postulated for CD146 in addition to its role as a cell adhesion molecule. Using cultured endothelial cells (HUVECs), Anfosso et al demonstrated an outside-in signaling pathway linked to CD146 engagement [8]. In this pathway, activation results in the tyrosine phosphorylation of a complicated pattern of proteins, including paxillin and p125^FAK^. Engagement of CD146 in endothelial cells recruits p59^fyn ^to the vicinity of CD146, as well as triggering a store-dependent Ca^2+ ^influx. Solvey and colleagues [1] found that CD146 binding led to a change in cellular permeability, actin distribution, and redistribution of NF-κB p50 to the nucleus.

The finding of CD146, an antigen widely accepted as an endothelial adhesion molecule, on peripheral blood lymphocytes, has yet to be explained. Furthermore, there has been no previous effort to characterize the nature of CD146+ T cells in the peripheral circulation. To this end, the current study was undertaken to understand the nature of CD146+ T cells through phenotypic and genotypic profiling of these cells.

## Results

### Identification of CD146 Positive Lymphocytes

**Figure 1:**
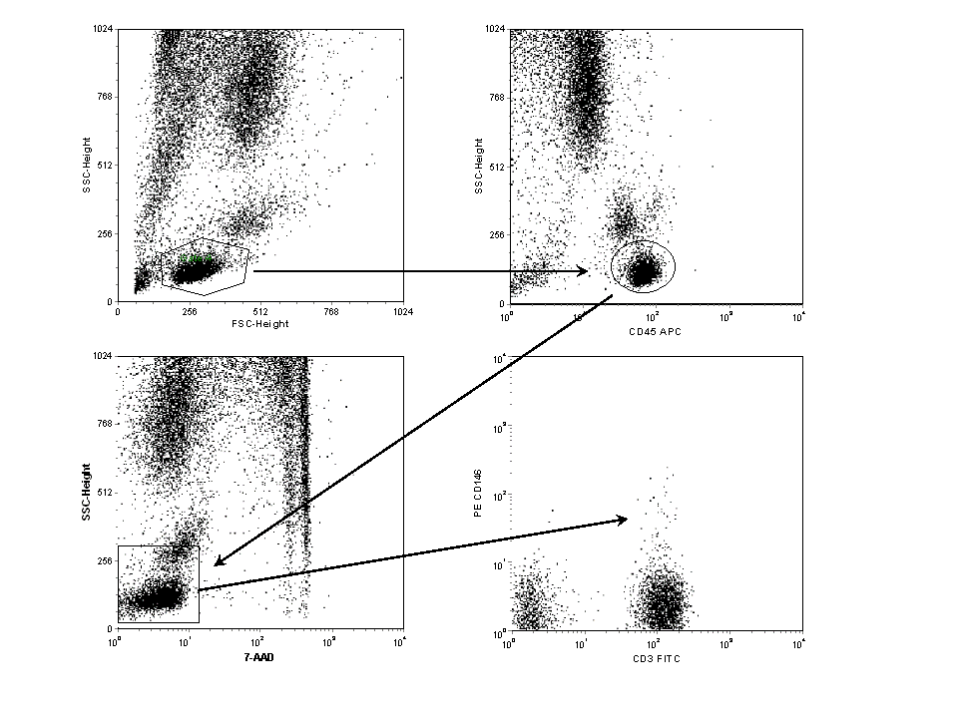
Example of the gating strategy used to identify CD146 positive T cells. Gates were set around lymphocytes, as defined by forward and side light scatter (upper left), CD45 bright-positive cells (upper right), and live cells determined as 7AAD-negative (lower left). All three gates were applied to the lower right dot plot displaying CD3 and CD146.

**Figure 2:**
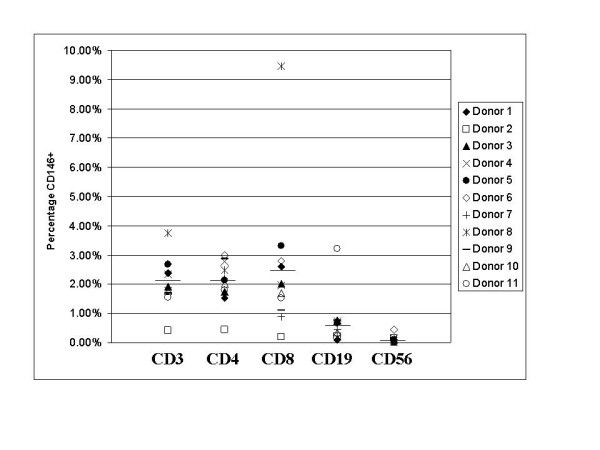
Expression of CD146 by various lymphocyte subsets in the peripheral blood of healthy volunteers. Bars indicate means for each subset. All cells were gated as described for Figure 1, with the addition of CD3 positive gating for CD4 and CD8, and CD3 negative gating for CD56.

Specimens of fresh peripheral blood from healthy individuals were stained with the monoclonal antibodies listed in the methods section and analyzed by flow cytometry. CD45/CD146 dual positive cells were consistently identified in peripheral blood samples from healthy individuals, and these were identified in lymphocyte subsets as illustrated in Figure 1. In most peripheral blood samples, the CD45+ CD146+ cells represented 1% or less of the mononuclear cells. Using a light scatter gate for lymphoid cells and viability gating, approximately 2% of the CD3 positive T cells were CD146 positive [%dual CD3+ CD146+/%CD3+] (2.09% [mean] ± 0.84% [SD], n = 10). Among the CD3, CD146 positive lymphocytes, both CD4 and CD8 subsets were present. In most, but not all, individuals the proportion of CD3+CD4+ CD146+ cells was similar to D3+CD8+CD146+ (Figure 2). The percentage of CD3+CD4+ cells expressing CD146 [i.e. %triple CD3+CD4+ CD146+/%dual CD3+CD4+] was 2.08% ± 0.73% (n = 10) and the percentage of CD3+CD8+ cells expressing CD146 [i.e. %triple CD3+CD8+ CD146+/%dual CD3+CD8+] was 2.50% ± 2.47%. The rather wide standard deviation on the latter was largely the result of a single patient who demonstrated 9.45% of CD8 cells expressing CD146. Exclusion of this high value yielded 1.80% ± 0.94% (n = 9) of the CD3+CD8+ cells expressing CD146.

The CD3, CD146 dual positive cells also expressed additional T cell markers CD2, CD5 and CD7, (data not shown) confirming that these cells were T lymphocytes rather than another cell type aberrantly expressing CD3. The use of pulse width measurements eliminated the possibility that these cells were doublets of CECs and T cells [9], and the use of a viability stain (7-AAD), as well as isotype controls, excluded the possibility that this staining was due to nonspecific binding of the monoclonal antibodies. The expected immunophenotype of CECs, CD45-CD3-CD146+ [10], represented an average of 0.047% of the nonerythroid peripheral blood cells (range 0.005% to 0.142%) in the donors studied.

Soluble CD146 (sCD146) has previously been demonstrated in culture supernatants of endothelial cells as well as in the plasma of patients with chronic renal failure. To investigate whether the CD146 present on cells in fresh peripheral blood was due to binding of soluble CD146 to these cells, peripheral blood mononuclear cells, as well as whole peripheral blood, were washed with PBS twice prior to staining as described above. No change in either the percentage of T cells positive for CD146 or in the intensity of CD146 staining was observed. While these data suggest that the CD146 detected on lymphocytes was intrinsic rather than extrinsic due to binding of soluble CD146, further evidence regarding the origin of lymphocytic CD146 was obtained by the *in vitro *activation experiments described later.

Other lymphocyte subsets, B cells and NK cells were examined for the presence of CD146. Since these cell types each comprise a far lower percentage of the lymphocytes in normal peripheral blood, larger numbers of cells (greater than 4 × 10^5^) were collected per list mode file for analysis. CD45+, CD19+, CD146+, 7AAD- cells represented 0.74% of CD45+, CD19+, 7AAD- B cells [i.e. %CD45+, CD19+ CD146+/%CD45+CD19+] (0.74% ± 0.86%, n = 10). NK cells were identified as CD45+, CD3-, CD16+, and CD56+. In fresh peripheral blood, an extremely low percentage (0.11% ± 0.13%, n = 10) of the NK cells were determined to be CD146+ [i.e. % CD3-CD16+CD56+CD146+/%CD3-CD16+CD56+]. These lymphocyte immunophenotyping data are summarized in Figure 2.

### Lack of coexpression of activation markers and leukocyte adhesion marker

**Figure 3:**
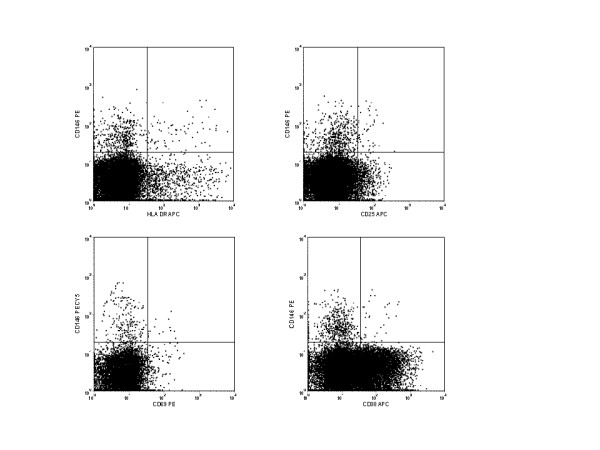
**A**. Representative histograms comparing CD146 staining on CD3+ T cells to HLA-DR, CD25, CD69, and CD38, other common markers of activation. Only a small number of cells stained with both markers, indicating that largely unique subsets were identified by each. In healthy individuals, the percentage of HLA-Dr + T cells expressing CD146 was 5.5% (± 1.99%; n = 5), the percentage of CD25+ T cell expressing CD146 was 8.36% (± 1.86%; n = 5), the percentage of CD69+ T cells expressing CD146 was 8.47% (± 3.08%; n = 5), and the percentage of CD38+ T cells expressing CD146 was 0.53% (± 0.19%; n = 4).

Studies were undertaken to determine if the expression of CD146 coincided with the expression of other well-defined activation markers of lymphocytes. Multicolor flow cytometry was used to examine coexpression of CD146 with CD25, CD69, CD38, and HLA-DR on CD3 positive cells in fresh peripheral blood. Representative histograms of some of these experiments are given in Figure 3. These data indicate that CD146+CD3+ cells are largely distinct, but not entirely unique, compared to other T cell subsets identified by different markers of activation.

**Figure 4:**
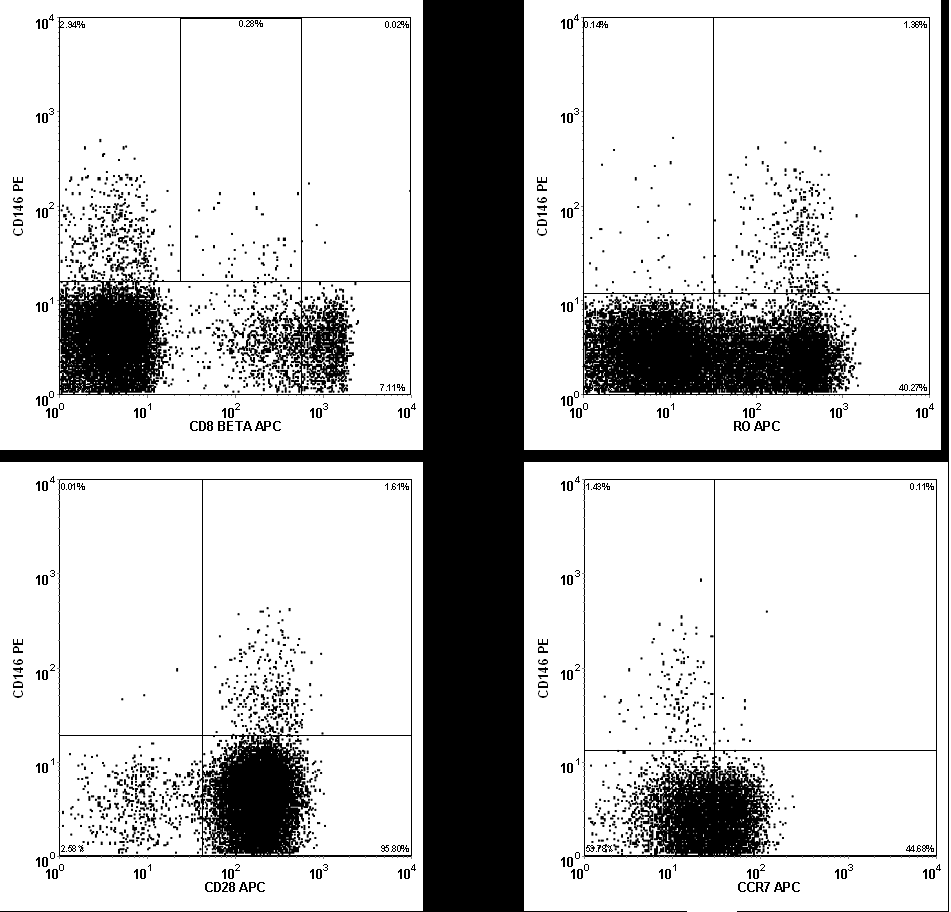
Representative histograms of CD3+ T cells stained with CD146 and CD8β, CCR7, CD45RO, and CD28. Note that the majority of the CD146+ T cells are CD28+, CD45RO+, CCR7-, and dim for CD8β.

The CD3, CD146 dual positive cells were further characterized by examination of additional markers of interest. This population of cells was found to be alpha/beta positive, gamma/delta negative, and predominantly positive for expression of CD28 (Figure 4). Since there is substantial homology between CD146 and CD166 [3], (the activated leukocyte cell adhesion molecule) CD3, positive cells were examined for the coexpression of these molecules. Very few cells demonstrated coexpression of these two molecules, suggesting that these two adhesion molecules identified substantially different T cell subsets.

### TCR Vβ Analysis

The CD3, CD146 dual positive cells were TCRαβ positive and TCRγδ negative. To ascertain whether CD3+CD146+ cells represented clonal populations of T lymphocytes, a panel of antibodies to various TCR vβ regions were used to stain both freshly isolated, and mitogen-stimulated, peripheral blood mononuclear cells from healthy individuals. The data obtained from both fresh and stimulated blood indicated that CD3+CD146+ T cells were polyclonal, with a number of different TCR vβ regions expressed, but none exclusively or even predominantly. Thus the CD146 positive T cells did not represent a clonal expansion.

### Lack of coexpression of primitive markers of lymphocytes

There was a virtual absence of CD34 and CD133 on CD45+ CD146+ lymphocytes. In a sampling of 5 healthy individuals, there was an average of 0.01% of the CD45+CD146+ cells expessing CD34, and 0.016% expressing CD133.

### Lack of coexpression of endothelial associated markers

CD3+ T cells staining for CD146 were negative for the expression of the endothelial markers CD51/61 and von Willebrand factor. The CD146+ T cells were also predominantly negative for CD31. This further indicates that the observed staining was not due to lymphocyte-endothelial cell doublets, as the only endothelial marker expressed on the T cells was CD146. CD31, which can be expressed on T cells as well as endothelial cells, displayed infrequent co-staining with CD146. In general, however, these two markers identified different subsets of T cells.

### Analysis of Chemokines, Chemokine Receptors, and Memory markers

A paucity of CCR7 expression was found on CD146+ CD3+ T cells in peripheral blood from healthy volunteers (Figure 4). This was consistent for both CD4 and CD8 T cells. No consistent positive or negative correlation was found between CD146 and CCR5, CXCR3, or CXCR5. Analysis of CD45RA and CD45RO revealed that CD3+ CD146+ T cells were almost entirely CD45RA negative and primarily CD45RO positive; consistent with a memory phenotype. The expression of CD62L was variable on both CD146 positive and negative T cells.

### In vitro activation of T cells

A previous report by Pickl et al [5] demonstrated CD146 expression could be induced by *in vitro *activation of T cells with mitogen stimulation. To reproduce, and extend, these observations, peripheral blood mononuclear cells were cultured *in vitro *in the presence of PHA, PWM, ConA, or IL-2 for varying lengths of time up to 5 days. In some studies, 5 μM carboxyfluorescein diacetate succinimidyl ester was added to the culture to study cell proliferation.

**Figure 5:**
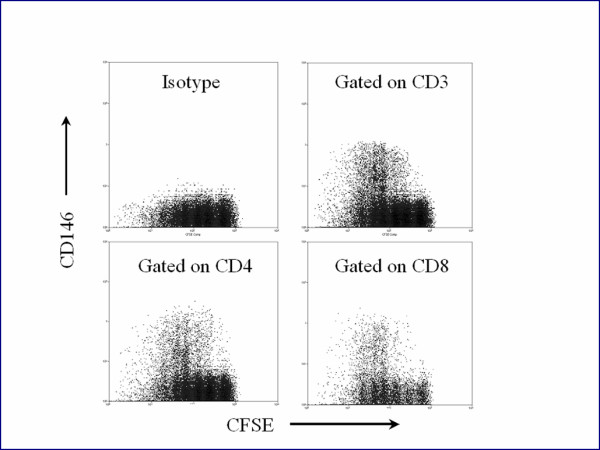
Mononuclear cells from the peripheral blood of a normal volunteer were incubated with carboxyfluorescein diacetatesuccinimidylester (CFSE) and PHA, as described in the text. CFSE fluorescence reduced with each cellular division. The cells were cultured a total of five days. The upper left panel displays CFSE staining versus a PE isotype control of CD3+ cells. The upper right panel shows staining of PECD146 versus CFSE of CD3+ T cells. Lower panels display CFSE vs CD4 and CD8. CFSE staining decreased with each successive division of the T cells, and CD146 expression increased.

These experiments demonstrated that mitogen stimulation increased the number of T cells expressing CD146 (approximately 4 to 10-fold) as well as increasing the mean intensity of the CD146 staining. The intensity of staining increased with every subsequent cell division up to the fifth division, as evidenced by the CFDASE costaining (Figure 5). Similar upregulation of CD146 expression on T cells was observed using IL-2 to activate the cells.

To determine if the increase in CD146 positive T cells observed above was due to expansion of preexisting CD146 positive cells or was due to *de novo *expression of CD146 on T cells that had previously been CD146 negative, cell sorting experiments were conducted. CD146 negative T cells were purified by flow cytometric cell sorting, and then cultured *in vitro *with mitogens, as above. In replicate experiments CD146 expression was induced by 72 hour mitogen stimulation in the sorted CD146 negative T cells.

### In vitro activation of B cells

Since there has been no report of CD146 expression on B lymphocytes, the observation that a very small percentage CD146 positive B cells in the circulation was unexpected. To determine if the CD146 expression on B lymphocytes could be upregulated in a manner similar to that observed on T cells, freshly isolated B cells were activated *in vitro *with CD40L and IL-4. Similar to T cells activated *in vitro*, the activated B cells displayed increased numbers of CD146+ cells after 5 days of activation (5 to 10 fold), and the intensity of CD146 staining increased compared to the fresh cells. CD146 negative B cells, sorted by flow cytometry from either fresh peripheral blood or from populations of B cells activated *in vitro*, revealed *de novo *expression of CD146 after 5 days of *in vitro *activation.

In fresh blood peripheral blood B cells, CD146 expression displayed only a slight overlap with CD27, CD69, and HLADR staining. After activation with CD40L, virtually all CD146+ B cells displayed co-staining with CD27, CD69, and HLA-DR.

### Adherence assays

**Figure 6:**
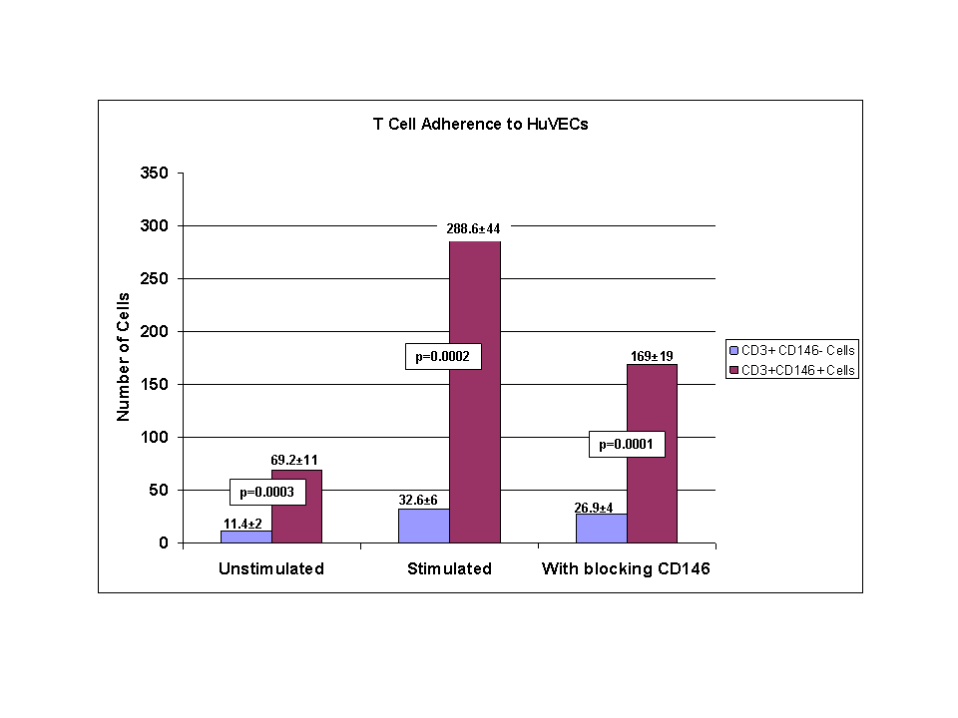
Adherence of T cells to activated endothelial monolayers. Cells were sorted by flow cytometry into two populations – one was CD3+CD146-, the other CD3+CD146-+. Cells were tested without stimuation, with PHA stimulation, and with CD146 blocking antibody following stimulation, as described in the methods. Data are shown as adhered cells per high powered field of light microscopy. The mean and standard deviations of 5 replicate experiments are shown. P values from paired t tests are shown.

In order to assess whether CD146 positive T cells exhibited an increased ability to adhere to endothelial cells, *in vitro *binding assays were conducted. Lymphocytes were sorted into CD3+CD146+ and CD3+CD146- populations, and some aliquots of these cells were activated *in vitro *with PHA or pokeweed mitogen. Both activated and unactivated cells were then incubated on monolayers of activated HUVEC cells. These experiments (Figure 6) revealed that activation of T cells substantially increased the number of cells capable of binding to the HUVECs and that, without stimulation, CD146+ T cells were more adherent than were CD146- T cells. The monolayers of HUVECs with attached T cells were also harvested and analyzed by flow cytometry (data not shown). These studies confirmed that the attached cells were T cells.

Blocking studies in which the activated T cells were preincubated with CD146 antibodies demonstrated a significant, but not complete, inhibition of the increased binding (Figure 6).

### Oligonucleotide microarray results

**Figure 7:**
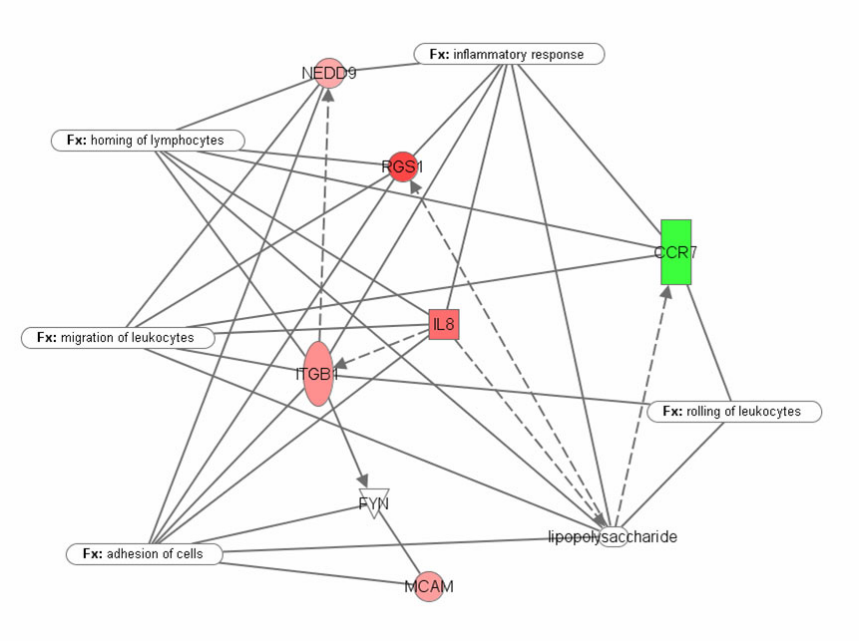
The microarray studies and analysis by Ingenuity™ (21) showed that CD+146 cells up-regulate a cluster of genes (red) that prepare them for the mechanisms of extravasation and destination to an inflammatory site. The green down-regulated gene (CCR7) indicates capability for the reverse migration, i.e. from peripheral tissues into vasculature or into lymphatics

Chip analysis of the RNA purified from the CD146 positive T cells and the CD146 negative T cells revealed substantial differences in the genes expressed between the two cell types. Using the predefined limits for significance (see Methods) a list of 104 probe sets comprising 84 unique genes was found. A list of the genes demonstrating the highest absolute log-fold changes in expression between the CD146+ T cells and the CD146- T cells, with the greatest statistical significance (FDR ≤ 0.005) is displayed in Table 1. The melanoma adhesion molecule, CD146, used as the basis for separating the two populations of T cells, ranked 38^th ^among genes preferentially expressed in CD146 positive T cells compared to the CD146 negative cells, with a 7-fold change. Dual specificity phosphatases displayed some of the highest differences, with DSP-4 having a 5-fold increase in the CD146+ T cells. Many of the genes preferentially expressed by CD146+ T cells play roles related to signal transduction/cell communication functions or are involved in the regulation of nucleobase, nucleoside, nucleotide, and nucleic acid metabolism. Among the genes preferentially expressed in the CD146 negative T cells were the CD8 beta 1 chain, nitric oxide synthase interacting protein, and CCR7. There were some observed phenotypic correlations with the genotyping profile (Figure 4). Staining for CCR7, for example, revealed very little expression on CD146+ T cells, and CCR7 displayed a 6.3-fold association with CD146 negative cells. Similarly, CD8beta was negatively associated with CD146 by gene expression studies, and immunophenotyping revealed the vast majority of CD146+ T cells were either CD4+ or CD8beta low expressors (Figure 4). Some genotypic observations, such as the association of CD86 with CD146+ cells, did not correlate directly with immunophenotypic observations. IPA analysis revealed a cluster of genes involved with the mechanisms of extravasation (Figure 7).

**Table 1:** List of 66 genes with the largest log fold changes between CD146 positive and CD146 negative T cells.

**Rank**	**Gene**	**Fold Increase (or decrease) CD3+CD146+/CD3+CD146-**	Function*	Process*
1	Dual specificity phosphatase 4	65	Protein phosphatase activity	Signal transduction/cell communication
2.	Regulator of G-protein signaling 1	41.8	GTPase activator	Signal transduction/cell communication
3	Nuclear receptor subfamily 4, group A member 2	35.7	Ligand-dependent nuclear receptor	Signal transduction/cell communication
4	v-jun sarcoma virus 17 oncogene homolog	22.5	_	_
5	Kruppel-like factor 10	22.3	Transcription factor activity	Regulation of nucleobase, nucleoside metabolism
6	Chromosome 10 open reading frame 128	20.6	Unknown	Unknown
7	Dual specificity phosphatase 1	19.9	Protein phosphatase activity	Signal transduction/cell communication
8	Interleukin 8	19.1	Cytokine	Immune response
9	RAR-related orphan receptor C	18.1	Ligand-dependent nuclear receptor	Regulation of nucleobase, nucleoside metabolism
10	Family with sequence similarity 46, member C	15	Unknown	Unknown
11	FBJ murine osteosarcoma viral oncogene homolog B	13.6	Transcription factor activity	Regulation of nucleobase, nucleoside metabolism
12	Pre-B-cell colony enhancing factor 1	12.3	Cytokine	Signal transduction/cell communication
13	A kinase (PRKA) anchor protein 2	11.9	Cytoskeletal anchor	Cell growth and/or maintenance
14	Pellino homolog 1	11.5	Receptor signaling complex scaffold activity	Signal transduction/cell communication
15	Chimerin1	11.1	GTPase activator activity	Signal transduction/cell communication
16	Solute carrier family 6, neurotransmitter transporter, GABA, member 13	10.7	Auxiliary transport protein activity	Transport
17	Tribbles homolog 1	10.4	Protein kinase activity	Signal transduction/cell communication
18	Integrin beta 1 (fibronectin receptor)	9.7	Receptor activity	Signal transduction/cell communication
19	Rho GTPase activating protein 10	9.6	GTPase activator activity	Signal transduction/cell communication
20	Phorbol-12-myristate-13-acetate-induced protein 1	9.5	Unknown	Apoptosis
21	Tumor necrosis factor alpha-induced protein 3	9.3	Transcription regulator activity	Regulation of nucleobase, nucleoside metabolism
22	CD83 antigen	9.2	Unknown	Immune response
23	DEAD (asp-Glu-Ala-Asp) box polypeptide 31	8.6	RNA binding	Regulation of nucleobase, nucleoside metabolism
24	Similar to RIKEN cDNA 1200014N16 gene	8.2	Unknown	Unknown
25	Cyclin L1	8.2	RNA binding	Regulation of nucleobase, nucleoside metabolism
26	Alpha thalassemia/mental retardation syndrome X-linked	7.9	_	_
27	Pvt1 oncogene homolog, MYC activator	7.7	_	_
28	SNF1-like kinase	7.7	Protein kinase activity	Signal transduction/cell communication
29	Basic helix-loop-helix domain containing class B, 2	7.6	Transcription regulator activity	Regulation of nucleobase, nucleoside metabolism
30	Phytoceramidase, alkaline	7.6	Hydrolase activity	Metabolism;energy pathways
31	BTG family, member 3	7.4	Regulation of cell cycle	Signal transduction/cell communication
32	Runt-related transcription factor 1	7.4	Transcription factor activity	Regulation of nucleobase, nucleoside metabolism
33	Phosphodiesterase 4D, c-AMP-specific	7.2	Phosphoric diester hydrolase activity	Signal transduction/cell communication
34	SKI-like	7.2	unknown	Signal transduction/cell communication
35	DEAD (asp-Glu-Ala-Asp) box polypeptide 59	7.2	RNA binding	Regulation of nucleobase, nucleoside metabolism
36	Dynamin 3	7.1	GTPase activity	Signal transduction/cell communication
37	Interferon-related Developmental regulator 1	7.1	Receptor binding	Signal transduction/cell communication
38	Bicaudal D homolog 1	7	Transporter activity	Transport
39	Melanoma cell adhesion molecule	7	Cell adhesion molecule	Signal transduction/cell communication
40	Clone IMAGE: 110218 mRNA sequence	6.9	_	_
41	Trophoblast-derived noncoding RNA	6.9	_	_
42	CDNA clone IMAGE: 4077090, partial cds	6.7	_	_
43	Hydroprostaglandin dehydrogenase 15-(NAD)	6.6		
44	PERP, TP53 apoptosis effector	6.5	Unknown	Apoptosis
45	Hypothetical protein MGC14376	6.4	Unknown	Unknown
46	Interleukin 1 receptor, type 1	6.2	Transmembrane receptor activity	Signal transduction/cell communication
47	Ring finger protein 10	6.1	Ubiquitin-specific protease activity	Protein metabolism
48	Protein phosphatase 2, regulatory subunit B(B56) gamma isoform	6	_	_
49	Protein tyrosine phosphatase, non-receptor type 13	6	Protein tyrosine phosphatase activity	Signal transduction/cell communication
50	v-ets erythroblastosis virus E26 oncogene homolog 1	5.9	Transcription factor activity	Regulation of nucleobase, nucleoside metabolism
51	DKFZP586A0522 protein	5.8	Methyltransferase activity	Metabolism; energy pathways
52	Similar to echinoderm microtubule associated protein like 5	5.8	_	_
53	Hypothetical protein FLJ43663	5.8	Unknown	Unknown
54	Deleted in lymphocytic leukemia, 2	5.8	Unknown	Cell growth and/or maintenance
55	CDNA clone IMAGE: 3542720	5.8	-	-
56	Tetratricopeptide repeat domain 8	5.6	Unknown	Cell growth and/or maintenance
57	DNA polymerase-transactivated protein 6	5.6	Unknown	Unknown
-9	Ribosomal protein S11	1/5.6	Ribosomal subunit	Protein metabolism
-8	Ribosomal protein S9	1/6.2	Ribosomal subunit	Protein metabolism
-7	Hypothetical protein MGC40157	1/6.2	Unknown	Unknown
-6	Chemokine (C-C motif) receptor 7	1/6.3	G coupled protein receptor	Signal transduction/cell communication
-5	ATPase, H+ transporting lysosomal 9 kDa, V0 subunit e	1/6.5	ATPase activity	Transport
-4	Ribosomal protein L28	1/6.6	Ribosomal subunit	Protein metabolism
-3	Nitric oxide synthase interacting protein	1/7.3	Transporter activity	Transport
-2	CD8 antigen, beta polypeptide 1	1/7.7	T cell receptor activity	Immune response
-1	Hypothetical protein FLJ14346	1/8.5	Unknown	Unknown

*from the Human Protein Reference Database

## Discussion

To date CD146 expression on peripheral blood lymphocytes has only been described anecdotally, often in the context of attempts to detect circulating endothelial cells. In such situations CD146 positive lymphocytes merely represent confounding factors in accurately enumerating CECs, and no attempt has been made to characterize these cells. Since a primary function of CD146 on endothelial cells is to serve as an adhesion molecule for homotypic binding of these cells, it is a reasonable hypothesis to suggest that CD146 may perform a similar role on lymphocytes. It is well know that lymphocytes extravasate through the endothelium, particularly at sites of inflammation, although the precise mechanisms involved in this process have yet to be completely elucidated. It is possible that CD146 expression on lymphocytes plays a role in this process by mediating the binding of activated lymphocytes to the endothelium.

The current data indicate a distinct immunophenotype and gene profile of the T lymphocytes expressing CD146. The expression of CD45RO together with the diminished of expression of CCR7 on CD3+ CD146+ T lymphocytes strongly suggests an effector memory role for these cells. This immunophenotype is consistent with cells predicted to home to inflamed, peripheral tissue sites of infection and thus generally supportive of the hypothesis offered above [11].

The gene profiling studies confirm the paucity of CCR7 on CD146+ T cells and further demonstrate that CD146+ cells have up-regulated a cluster of genes involved with adhesion, migration, homing, and ultimately inflammation as indicated in Ingenuity's Knowledge Database. The negative association of CD8β with CD146 expression is also of note in the gene profiling studies. While the majority of CD3 cells expressing CD146 were CD4+ and CD8-, all individuals tested did have CD3+CD8+ cells expressing CD146. Those CD8 T cells expressing CD146 were predominantly CD8β-low. [12] reported CD28+ CD8β-low, CD45RO+ lymphocytes displayed an activated, effector memory phenotype, consistent with the present data.

The current data indicate that CD146 + T cells adhere better than CD146- T cells to HuVEC endothelial monolayers in vitro, and that this binding is at least partially blocked through the use of CD146 antibodies. This enhanced ability of CD146+ T cells to bind to endothelium is also consistent with the proposed role for these cells in vivo. However, these data are at best suggestive, and much work remains to be done in order to demonstrate if, and how, CD146 T cells extravasate. Anecdotal observations by Pickl and colleagues [5] found elevated numbers of CD146+ T cells in the synovial fluid of patients with rheumatoid arthritis as well as in skin lesions of delayed-type hypersensitivity reactions. Preliminary studies from our group confirm the presence of elevated number of CD146+ T cells in inflammatory synovial fluids (data not shown).

The lack of correlation of the expression of CD146 with other common markers of lymphocyte activation (CD69, CD25, and HLA-Dr) is somewhat unexpected. The CD146+ T cell thus represent a distinct population of activated cells in the peripheral circulation that have heretofore not been characterized.

In addition to its role as a cell adhesion molecule, CD146 has also been found to be associated with functional changes in endothelial cells. Notably, CD146 has been demonstrated to be involved in a complex outside-in signaling pathway [2]. In endothelial cells, engagement of CD146 by monoclonal antibody initiates calcium mobilization leading to tyrosine phosphorylation of Pyk2, p130^Cas^, FAK, and paxillin. Solvey and colleagues [1] confirmed many of these findings and found that CD146 binding led to a change in cellular permeability, actin distribution, and redistribution of NF-κB p50 to the nucleus. The current data indicate that a number of genes involved in signal transduction and cell communication are upregulated in the CD146 positive T cells. Given the previous reports, it is reasonable to postulate that engagement of CD146 on the surface of T cells might also lead to outside-in signaling pathways in lymphocytes. In endothelial cells these pathways lead to changes in permeability and cytoskeletal rearrangement – pathways, if also present in lymphocytes, would likely be necessary to facilitate transendothelial migration.

## Conclusion

The present data show that CD146 is an inducible activation antigen on both B and T lymphocytes and small populations of these cells are present in the peripheral circulation of healthy individuals. The CD146+ T cells have an effector memory phenotype, demonstrate up-regulation of a cluster of genes involved with adhesion, migration, homing, and inflammation, and have enhanced binding to endothelial monolayers in vitro. All of these characteristics are consistent with cells involved in inflammatory responses. Based on the polyclonal nature of the CD146+ T cells in the peripheral circulation, we speculate that these represent a small pool of cells primed for extravasation in response to inflammatory stimuli.

## Methods

### Collection of blood

Peripheral blood was collected by routine venipuncture from healthy volunteers. Blood draws were obtained under a protocol approved by the National Heart, Lung, and Blood Institute Institutional Review Board. Acid citrate dextrose (ACD) or sodium heparin was used as the anticoagulant.

### Immunofluorescence staining

Stimulated or freshly isolated peripheral blood mononuclear cells (PBMCs) were washed and then surface stained with directly conjugated antibodies to CD146 and other surface markers (isotype matched, control monoclonal antibodies, or mouse antihuman CD2, CD3, CD4, CD7, CD8, CD8β CD10, CD14, CD19, CD20, CD25, CD28, CD29, CD31, CD34, CD38, CD45, CD45RA, CD45RO, CD62L, CD69, CD83, CCR7, CCR5, CXCR5, CXCR3, and HLA-DR monoclonal antibodies (all from Becton Dickinson Immunocytometry Systems, San Jose, CA) according to the manufacturer's instructions. FITC-CD166 was purchased from Ancell (North Bayport, MN) and APC-CD133 from Miltenyi Biotec GmbH (Auburn, CA). Additionally, PE-conjugated CD146 purchased from Chemicon was used in some experiments to compare to the BD antibody. Stained cells were washed three times with 1% BSA in PBS, pH 7.2, and then 7AAD (Beckman Coulter, Hialeah, FL) was added to stain dead cells. The cells were analyzed within 15 minutes after addition of 7AAD. In some experiments with fresh peripheral blood not requiring viability staining, a stain-lyse method was followed using FACSLyse (BDIS) as per the manufacturer's instructions.

T cell vβ usage by CD146 positive cells was examined using monoclonal antibodies to various vβ families in immunophenotypic studies. The following antibodies were purchased from Beckman Coulter (Hialeah, FL): vβ2, 3, 5.1, 5.2, 5.3, 7, 8, 9, 11, 12, 13.1, 13.6, 14, 16, 17, 20, pan αβ, and pan γδ.

### Flow Cytometric Immunophenotyping and cell sorting

Flow cytometric analysis was performed using a DakoCytomation CyAn (DakoCytomation, Fort Collins, CO) or a FACSCalibur (Becton Dickinson, San Jose, CA). Fluorescein isothiocyanate (FITC) or carboxyfluorescein diacetate succinimidylester (CFDASE), phycoerythrin (PE), PE-Texas Red, &-aminiactinimmycin-D (7-AAD [for viability staining]), PE-Cy7, allophycocyanin (APC) and APC-Cy7 were used as the fluorophores. At least 50,000 live CD3^+ ^lymphocytes were collected on each sample for analysis. Gates were set using light scatter and for live cells using 7-AAD exclusion.

Cell sorting experiments were performed on cells stained as described. Both the FACSAria (Becton Dickinson) and the MoFlo (Dako Cytomation) cell sorters were used for these experiments. Pulse width was used to eliminate doublets on all sorts, to eliminate the possible isolation of endothelial cell-lymphocyte duplexes (derived from 9). Populations of cells that were sorted were: CD45+CD3+CD146+; CD45+D3+CD146-; CD45+CD19+CD146+; and CD45+CD19+CD146-. A minimum of 3,000 cells were sorted for the gene profiling studies, and a minimum of 100,000 were sorted for the *in vitro* activation studies. CD146 positive T cells were obtained from five individuals, and CD146 negative T cells were examined from three of these five. B cell subpopulations were sorted from another 3 healthy individuals.

### T cell culture and activation

Peripheral blood mononuclear cells (PBMCs), obtained by density gradient separation, were washed and resuspended at a density of 0.5 × 10^6 ^cells per mL in RPMI 1640 medium containing 10% FCS (HyClone, Logan, UT), 25 mM Hepes, 2 mM Lglutamine, 100 IU/mL of penicillin, 100 μg/mL streptomycin, and 5 μM 2 mercaptoethanol. T cell activation was achieved by the addition of either phytohemagglutinin (PHA), concannavalin A (Con A), or pokeweed mitogen (PWM) (Sigma, St. Louis, MO) at 5 μg/mL, as described previously [5]. After 3 days cells were washed twice and resuspended in media with 10 μl/mL IL-2 (R&D, Minneapolis, MN) without further stimulation. Cells were passaged every 2 to 3 days, replenishing IL-2 with every passage. In some experiments, CFDASE was added to the cells to examine proliferation, as described below.

T cell culture and activation studies were also conducted on CD3+CD146cells sorted by flow cytometry. These were performed as described above to determine if CD146 expression *in vitro *could emanate *de novo *from a starting population of cells that were CD146 negative.

### B cell culture and activation

B cells were expanded from healthy donor PBMCs using CD40 ligand-transfected NIH3T3 cells (t-CD40L) as previously described [13,14] Briefly, 2 × 10^5 ^irradiated (75 Gy) t-CD40L cells (kindly provided by Dr M. Nishimura, University of Chicago) were plated into 6-well plates (Costar, Cambridge, MA) and cultured overnight at 37°C in 5% CO2. The following day, media were removed, and 4 × 10^6 ^to 6 × 10^6 ^PBMCs suspended in 3 mL Iscove-modified Dulbecco medium (IMDM) (Cellgro; Mediatech, Herndon, VA) supplemented with 10% pooled human serum, IL-4 (200 U/mL; PeproTech USA, Rocky Hill, NJ), and clinical grade CSA (5.5 × 10^7 ^M; Novartis, Basel, Switzerland) were added to each well and cultured at 37°C in 5% CO2. Approximately every 3 to 4 days, expanded B cells were washed and then transferred onto freshly prepared irradiated t-CD40L cells in cytokine replenished medium. Expanded B cells were checked CD146 expression by flow cytometry on Days 7–8.

### Carboxyfluorescein diacetate succinimidylester cell labeling and culture conditions

To examine CD146 expression as a function of cell division, PBMCs were labeled with CFDASE during growth *in vitro *for 5 days (method reviewed in 15). Cells were stimulated with PHA as described above. Prior to incubation, PBMCs were washed and resuspended at a density of 2 × 10^7^cells per mL in PBS. An equal volume of 5 μM carboxyfluorescein diacetate succinimidyl ester (CFDASE; Molecular Probes, Inc., Eugene, OR) in PBS was added, and the cells were gently mixed for 15 min at 37°C. Unbound CFDASE, or the deacetylated form, CFSE, was quenched by the addition of an equal volume of fetal bovine serum (FBS). Analysis of cells immediately following CFSE labeling indicates a labeling efficiency that exceeded 99%, and all cells remain labeled for at least 5 days.

The labeled cells were washed two times in PBS and resuspended at 1 × 10^6 ^cells per mL in RPMI 1640 medium containing 10% FCS (HyClone, Logan, UT), 25 mM Hepes, 2 mM L-glutamine, 100 IU/mL of penicillin, 100 μg/mL streptomycin, and 5 μM 2 mercaptoethanol. Labeled cells were plated at 1 × 10^5 ^cells per well in round bottom 96 well microtiter plates, and T cell activation was achieved by the addition of phytohemagglutinin (PHA) (Sigma) or Con A (Sigma) at 5 μg/mL.

### Endothelial Cell Culture

HUVEC (human umbilical vascular endothelial cells, Cambrex) were cultured in EGM™ 2 MV medium (Cambrex) in BD Falcon Tissue Culture flasks and subcultured at 50 to 70% confluence before harvesting for analysis or culture in 24-well plates for adherence assays.

### Adherence Assays

Peripheral blood mononuclear cells prepared and stained, as described above, were sorted into CD3+CD146+ and CD3+ CD146- populations. These populations were suspended in 600 μl RPMI (plus 10% FCS) and incubated either with or without 5 μg/mL PHA for 72 hours at 37°C with 5% CO_2 _prior to the adherence assay (modified from 16). The cells were washed twice with 2 mL RPMI (plus 10% FCS) and finally resuspended at 1 × 10^6 ^cells/mL in RPMI (plus 10% FCS). HUVEC cells, grown to confluency in 24-well plates (with or without Transwell inserts), were used to assess adherence. HUVECs were activated by exposure to 1 ng/ml IL-1B in medium (RPMI/I0% FCS) for 4 hours at 37°C and then washed once with medium immediately before addition of T cells. [17]. The medium was aspirated from each well and replaced with 500 μl of either CD146+ or CD146- T cells (1 × 10^6 ^cell/ml). Each test was performed in duplicate. After 1 hour incubation at 37°C, loosely adherent cells were removed by washing the monolayers 3 times with 500 μl RPMI. The numbers of adherent lymphocytes were enumerated using light microscopy by counting small rounded cells atop the endothelial monolayers per high power field. A minimum of 10 fields were counted in each experiment and averaged.

### Antibody Blocking Studies

Experiments were conducted to determine the effect of blocking the lymphocyte CD146 surface antigen on the binding of these cells to activated endothelial monolayers. Lymphocytes, at a concentration of 10 × 10^6^cells/mL in culture media were incubated with anti-CD146 (clone P1H12, BD Pharmingen, San Jose, CA) (20 μg/10^6 ^cells) for 45 min at 37°C. The cells were washed once and resuspended at 1 × 10^6 ^cells/mL before addition to the endothelial monolayers. The adherence assays were then performed as described above.

### Isolation of RNA from Flow Sorted Cells

Cells were collected in lysis buffer containing guanidinium thiocyanate and total RNA was extracted using RNAqueous micro RNA isolation kit (Ambion, Austin, TX) following the manufacturer's directions. In brief, cell lysate was mixed with ethanol and applied to a silica based filter that selectively binds RNA. Genomic DNA from the eluted RNA was removed by DNase treatment. The concentration of the isolated RNA was determined using a nanodrop ND-1000 spectrophotometer. Quality and integrity of total RNA was assessed on an Agilent 2100 bioanalyzer.

### Amplification of RNA and Gene Expression studies

Linear T7 based RNA amplification was carried out on 10 ng of the isolated total RNA using Riboamp OA 2 round amplification kit as suggested by the manufacturer (Arcturus, Mountain View, CA). Briefly, total RNA was incubated with oligo dT/T7 primers and reverse transcribed into double stranded cDNA. *In vitro *transcription of the purified cDNA was performed using T7 RNA polymerase at 42°C for 6 hours. The amplified RNA was purified and subjected to a second round of amplification with biotin labeling using Affymetrix's IVT labeling kit following the manufacturer's directions. The yield and integrity of the biotin labeled cRNA were determined using Nanodrop ND-1000 spectrophotometer and Agilent 2100 bioanalyzer. Biotin labeled RNA (20 μg) was fragmented to ~200 bp size by incubating in 200 mM Tris-Acetate pH 8.2, 500 mM KOAc, MgOAc fragmentation buffer for 35 min at 94°C prior to hybridization. Fragmented cRNA was assessed for fragment size on Agilent 2100 bioanalyzer and hybridized to Affymetrix U133A plus 2.0 chips for 16 hours, and the hybridized gene chips were washed and stained on an Affymetrix fluidics station.

### Microarray Data Processing and Statistical Analysis

Affymetrix GCOS version 1.2 software was used to calculate signal and present call values which were stored in the NIHLIMS, a database for storage and retrieval of chip data maintained at NIH. Data were statistically analyzed using the MSCL Analyst's Toolbox [18] and the JMP statistical software package (SAS, Inc, Cary, NC). The results for 8 chips were retrieved and signal values were subjected to an adaptive variance-stabilizing, quantile-normalizing transformation termed "S10" [19]. This transform both normalizes between chips over the full data range, and makes the variance of replicates nearly uniform over expression level. This transform approximates a common logarithm transform over the central working range of the assay (data not shown), and thus differences of S10-transformed data values can be interpreted as log ratios or simply "log-fold changes". A major advantage of this approach over the ordinary log-ratio is that changes in S10-transformed values have a uniform variance over the full expression scale. Visualization of the global results and detection of possible outliers among the 8 samples was facilitated by principal components analysis (PCA) of the transformed data and presentation in bivariate plots of low-order principal components.

### Selection of Significant Genes

To quantify the significance of gene expression differences, a one-way, two-level ANOVA was applied comparing activated (n = 5) to resting (n = 3) T-cells. The p-value for differences between the two tissues was collected for each of the 54,675 Probe Sets. To address the multiple comparisons problem, the false discovery rate (FDR) [20] was controlled. Log fold-changes were computed as the difference between average values for the two groups. Probe sets with greater than or equal to 5-fold change in either direction, less than or equal to 0.5% FDR (i.e. 0.005) and with greater than 50% of samples in at least one group having a "present call" were identified as highly significant and subjected to further analysis. Data were subsequently used for Ingenuity Pathway Analysis (IPA) [21] to model relationships among genes and proteins and to construct putative pathways. The Ingenuity Pathways Knowledge Base contains numerous modeled relationships among proteins, genes, complexes, cells, tissues, drugs, and diseases. These modeled relationships rely upon data from various primary literature sources, including peer-reviewed journal articles, review articles, and textbooks as well as other types of content, including NCBI databases (EntrezGene, RefSeq, OMIM disease associations), Gene ontology annotations, normal gene expression for various tissues from the Genome Novartis Foundation Body Atlas, KEGG and LIGAND metabolic pathways, and cell signaling pathways.

## Authors' contributions

MFE performed the lead role in all experimental work and helped to write the manuscript; SSK performed initial flow cytometric experiments; NR performed RNA extractions and amplifications; YT performed B cell activation studies; JB, JJB, and PJM performed the gene chip analysis and pathway analyses; MAS and RD assisted with experimental design and data interpretation; JPM participated in the design of experiments, coordinated the study, and participated in the writing of the manuscript. All authors have read and approved the final manuscript.
